# Nutcracker Syndrome: A Rare Cause of Hematuria

**DOI:** 10.1100/tsw.2006.144

**Published:** 2006-06-30

**Authors:** Emmanuel C. Gorospe, Michael O. Aigbe

**Affiliations:** ^1^ The Children's Nephrology Clinic, Las Vegas, NV, USA; ^2^ Department of Pediatrics, University of Nevada School of Medicine, Las Vegas, USA

**Keywords:** Nutcracker syndrome, hematuria

## Abstract

Nutcracker syndrome is the compression of the left renal vein between the aorta and superior mesenteric artery. It is a rare cause of hematuria which results from the rupture of congested renal veins into the collecting system.

Nutcracker syndrome refers to the compression of the left renal vein between the aorta and superior mesenteric artery[1]. This compression causes venous hypertension, which results in the formation of renal venous collaterals and subsequent rupture of the septum between the veins and the collecting system, manifesting as hematuria[2]. This CT angiogram (Fig. 1) is from a 7-year-old male who was referred for a 6-month history of intermittent gross hematuria. He had no preceding infection, abdominal trauma, or renal colic. The patient's family and past medical histories were unremarkable. Physical findings were normal. Complete blood count, routine clinical chemistry, complement serology, anti-GBM antibodies, and bleeding parameters were within normal range. Urine analysis yielded only numerous red blood cells per high-power field. Initial ultrasound and cystoscopy failed to detect any renal, ureteral, or bladder anomaly. A biopsy was performed, which also showed no structural pathology on light and electron microscopy. Immunofluorescence microscopy was negative for immune reactants. Nutcracker syndrome is a rare cause of hematuria. It should be suspected in cases of persistent hematuria in the absence of nephrolithiasis, renal parenchymal disease, or any medical condition that may present with hematuria.

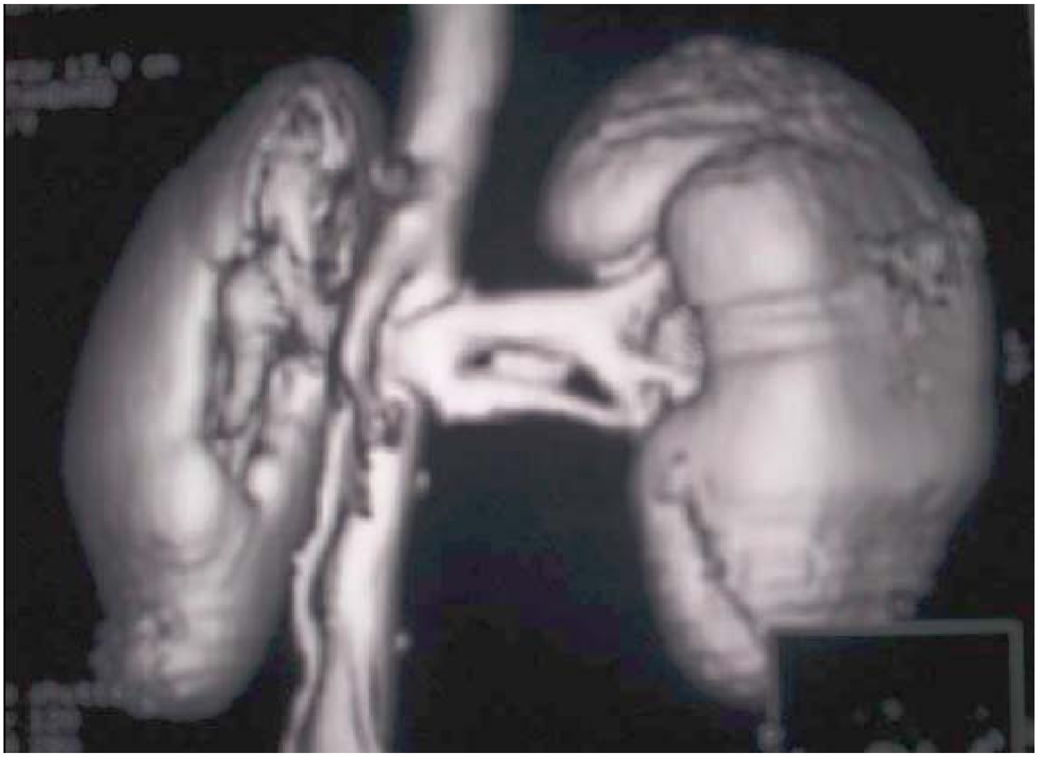

**FIGURE 1.** Multiplanar CT angiogram showing compression of the left renal vein between the aorta and superior mesenteric artery.

## References

1. El Sadr, A.R. and Mina, A. (1950) Anatomical and surgical aspects in the operative management of varicoceles. *Urol. Cutaneous Rev*. **54**, 257–267.
2. Lopatkin, N.A., Morozov, A.V., and Lopatkina, L.N. (1978) Essential renal haemorrhages. *Eur. Urol*. **4(2),** 115–119.
